# Rapidly Measured Indicators of Recreational Water Quality Are Predictive of Swimming-Associated Gastrointestinal Illness

**DOI:** 10.1289/ehp.8273

**Published:** 2005-09-01

**Authors:** Timothy J. Wade, Rebecca L. Calderon, Elizabeth Sams, Michael Beach, Kristen P. Brenner, Ann H. Williams, Alfred P. Dufour

**Affiliations:** 1 National Health and Environmental Effects Research Laboratory, Human Studies Division, U.S. Environmental Protection Agency, Research Triangle Park, North Carolina, USA; 2 Parasitic Diseases, Centers for Disease Control and Prevention, Atlanta, Georgia, USA; 3 National Exposure Research Laboratory, U.S. Environmental Protection Agency, Cincinnati, Ohio, USA

**Keywords:** bathing beaches, cohort studies, diarrhea, gastrointestinal diseases, Great Lakes Region, recreational water, swimming, water quality

## Abstract

Standard methods to measure recreational water quality require at least 24 hr to obtain results, making it impossible to assess the quality of water within a single day. Methods to measure recreational water quality in ≤ 2 hr have been developed. Application of rapid methods could give considerably more accurate and timely assessments of recreational water quality. We conducted a prospective study of beachgoers at two Great Lakes beaches to examine the association between recreational water quality, obtained using rapid methods, and gastrointestinal (GI) illness after swimming. Beachgoers were asked about swimming and other beach activities and 10–12 days later were asked about the occurrence of GI symptoms. We tested water samples for *Enterococcus* and *Bacteroides* species using the quantitative polymerase chain reaction (PCR) method. We observed significant trends between increased GI illness and *Enterococcus* at the Lake Michigan beach and a positive trend for *Enterococcus* at the Lake Erie beach. The association remained significant for *Enterococcus* when the two beaches were combined. We observed a positive trend for *Bacteroides* at the Lake Erie beach, but no trend was observed at the Lake Michigan beach. *Enterococcus* samples collected at 0800 hr were predictive of GI illness that day. The association between *Enterococcus* and illness strengthened as time spent swimming in the water increased. This is the first study to show that water quality measured by rapid methods can predict swimming-associated health effects.

Swimming in coastal waters is a favored pastime in the United States. In a survey of > 75,000 households, 42% of respondents ≥ 16 years of age, equivalent to approximately 89 million individuals, reported swimming in recreational waters annually (National Survey on Recreation and the Environment 2000–2002). Such waters are often contaminated by human sewage as a result of discharges or overflows [U.S. Environmental Protection Agency (EPA) 2001]. Swimming in fecally contaminated recreational waters has consistently been associated with gastrointestinal (GI) illness (Pruss 1998; Wade et al. 2003). The incidence of illness attributable to recreational water exposure appears to be increasing. The Centers for Disease Control and Prevention (CDC) reported 21 recreational water outbreaks in 2000, more than any single previous year since systematic surveillance began (Lee et al. 2002). The Natural Resources Defense Council (Dorfman 2005) reported that there were more beach closings and advisories in 2000 than in any previous year; 85% of these closings and advisories were due to bacteria levels that exceeded standards.

Because of the great diversity of pathogenic microorganisms transmitted by contaminated water and the difficulty and cost of directly measuring all microbial pathogens in environmental samples, organisms that may indicate the presence of sewage and fecal contamination (indicator organisms) are often used for monitoring and regulation of recreational and drinking waters. Indicator organisms are common inhabitants of the intestinal tract of warm-blooded animals. They are found in fecal material at high concentrations and are easier to measure in the environment than are pathogens. Although indicator organisms do not cause illness under normal conditions, they represent a measure of fecal contamination. Human sewage is a source of fecal contamination and also is known to contain pathogenic micro-organisms (Griffin et al. 2003; Jones 2001; Madore et al. 1987). Direct and indirect exposure to sewage has been associated with illness (Alexander et al. 1992; El-Sharkawi and Hassan 1982; Fleisher et al. 1996; Khuder et al. 1998; Mac Kenzie et al. 1994; Yamamoto et al. 2000).

Current recreational water-quality guidelines are based on studies conducted in the 1970s and 1980s (Cabelli et al. 1975, 1979, 1982; Dufour 1984). The currently recommended bacterial indicators are based on microbiological methods that involve culturing fecal indicator bacteria, such as *Enterococcus* spp. or *Escherichia coli*, and counting the colony-forming units. One shortcoming of these methods is that the bacteria require at least 24 hr to grow visible colonies, making it impossible for beach managers to assess the quality of water on the day of sample collection.

Because microbial water quality can change rapidly (Boehm et al. 2002), guidelines based on indicator organisms that require 24 hr to develop are likely to result in both unnecessary beach closings and the exposure of swimmers to poor-quality water. A recent study estimated that up to 40% of beach closures are in error (Kim and Grant 2004).

In 2000, Congress passed an amendment to the Clean Water Act, the Beaches Environmental Assessment and Coastal Health (BEACH) Act (2000). Among other provisions, the BEACH Act required the U.S. EPA to conduct research to provide the support of new criteria for recreational waters. Methods have been developed to measure microorganisms more rapidly. A modified version of polymerase chain reaction (PCR), quantitative TaqMan PCR (QPCR; Applied Biosystems, Foster City, CA), has been developed to quantify indicator bacteria in recreational waters (Santo Domingo et al. 2003) in ≤ 2 hr. Because these methods provide a faster assessment of water quality, they have the potential to significantly reduce illnesses resulting from exposure to recreational waters and also to reduce errors in beach closings or public notifications.

In 2003, we conducted the first in a series of studies designed to evaluate the ability of QPCR to predict health effects of recreational-water exposure. Secondary goals were to evaluate specific study design and analytical methods, such as methods for averaging indicator values, assignment of exposure measures to swimmers, and swimming definitions.

## Materials and Methods

We conducted a prospective cohort study of beachgoers at two beaches in the Great Lakes region. One beach was located in the Indiana Dunes National Lakeshore, in Indiana, on Lake Michigan (beach A), and the second was located near Cleveland, Ohio, on Lake Erie (beach B). The study consisted of a health survey of beachgoers and water-quality evaluation.

The beaches were selected specifically because they were affected by discharges from waste treatment plants. The sources of fecal contamination affecting beach A are waste-water treatment plant effluents from at least four communities that collectively contribute about 16 million gallons per day to small streams. The streams are tributaries of Burns Ditch, which empties into Lake Michigan approximately 2 miles east of the beach. Beach B is a short distance west of metropolitan Cleveland, Ohio. The beach is potentially affected by sewage treatment plant discharges into Lake Erie to the east and west. An outfall about 7 miles to the west discharges 6.5 million gallons of wastewater per day. Within 5 miles to the east of the beach, two other wastewater treatment plants discharge about 40 million gallons of treated sewage per day.

The study design, questionnaires, and materials were reviewed and approved by an Institutional Review Board for the CDC. All participants provided verbal informed consent before enrollment. We complied with all applicable ethical requirements, in accordance with all federal regulations for the protection of human subjects, in conducting this study.

### Beachgoer health surveys.

The health survey was administered in three parts: enrollment, beach interview, and telephone interview. Interviewers approached beachgoers on weekends and holidays during the summer. Beachgoers who agreed to participate provided verbal informed consent and returned to complete the beach interview as they left the beach. An adult (≥ 18 years of age) answered questions for other household members. The beach interview included questions about demographics, swimming and other beach activities, consumption of raw or undercooked meat or runny eggs, chronic illnesses, allergies, acute health symptoms in the past 48 hr, contact with sick persons in the past 48 hr, other swimming in the past 48 hr, and contact with animals in the past 48 hr. The telephone interview was conducted 10–12 days after the beach visit, and an adult ≥ 18 years of age answered questions for other household members who visited the beach. The telephone interview consisted of questions about health symptoms experienced since the beach visit, and other swimming- or water-related activities, contact with animals, and consumption of high-risk foods since the beach visit. Bilingual (English–Spanish) interviewers were available. Interviews were conducted at beach A between 1 June 2003 through 3 August 2003 and at beach B between 2 August 2003 and 14 September 2003.

Although respiratory, ear, eye, and skin rash symptoms were also evaluated, we present results only for GI illness. GI illness was defined as any of the following: diarrhea (three or more loose stools in a 24-hr period), vomiting, nausea and stomachache, and nausea or stomachache that affect regular activity (inability to perform regular daily activities). This definition of GI illness is consistent with definitions used in recent studies (Colford et al. 2002; Payment et al. 1991, 1997).

### Water sample collection and analysis.

Water samples were collected on each study day. Three times a day (0800 hr, 1100 hr, and 1500 hr), two water samples were collected at beach A along each of three transects perpendicular to the shoreline, one in waist-high water (1 m deep) and one in shin-high water (0.3 m deep). A representation of the sampling locations and additional details of the sampling protocol have been described previously (Haugland et al. 2005). Transects were located ≥ 60 m apart to include the area used by most beachgoers. Water samples were collected at beach A on weekends and holidays during the period from 31 May 2003 through 3 August 2003. Samples also were collected three times a day at nine beach B locations. Because jetties divided the beach and prevented free circulation of water, additional samples were collected to characterize the beach (Haugland et al. 2005). Samples were kept on ice at 1–4°C during the time before analysis.

A detailed description of sample preparation and QPCR analysis for *Enterococcus* spp. has been described elsewhere (Haugland et al. 2005). Primers and probes for the *Bacteroides* analyses were conducted as described by Dick and Field (2004), and analyses were conducted using conditions described by Haugland et al. (2005). Additional details regarding the estimation of cell equivalents have also been described (Applied Biosystems 1997). In brief, we used QPCR to detect and quantify *Enterococcus* and *Bacteroides* in water samples based on the collection of these organisms on membrane filters, extraction of their total DNA, and PCR amplification (i.e., a process whereby the quantity of DNA is doubled in each cycle of amplification) of a genus-specific DNA sequence using the TaqMan PCR product detection system. The reactions were performed in a specially designed state-of-the-art thermal cycling instrument (SMART Cycler TD System, Cepheid, Sunnyvale, CA) that automates the detection and quantitative measurement of the fluorescent signals produced by probe degradation during each cycle of amplification. Cell equivalents were estimated by comparing the cycle threshold to standard samples containing a known quantity of the target organism cells. If no threshold was achieved after 45 cycles, the sample was considered below the limit of detection. Because a separate set of calibrator reactions was conducted for each test sample, the limit of detection (LOD) can vary from sample to sample. This process has been described previously (Applied Biosystems 1997; Haugland et al. 2005). Water samples were filtered at the local laboratory (Great Lakes Scientific, Inc., Stevensville, MI, and Cuyahoga County Sanitary Engineering Division, Cleveland, OH), and filters were shipped on dry ice to the contract laboratory (EMSL Analytical Inc. Laboratory, Westmont, NJ) for QPCR analysis. Results for the QPCR analyses are expressed as QPCR cell equivalents (QPCRCE) per 100-mL volume.

### Data analysis.

We created two variables to represent exposure to indicator organisms: an average of all measures collected by day, and an average of measures specific to day and reported swimming location. The base 10 log (log_10_) of the geometric mean (the mean of the log_10_ of the count) was used for averaging results. Measures below the LOD were assigned values using maximum likelihood, assuming a log-normal distribution (El-Shaarawi and Viveros 1997). Quantile–quantile plots confirmed the approximate log-normal distribution of the water-quality measures, which are often approximately log-normally distributed (El-Shaarawi 1989; El-Shaarawi and Viveros 1997; Noble et al. 2003). We defined swimming in three ways: “any contact” included anyone reporting contact with water; and “body immersion” and “head immersion” included swimmers who reported a minimum of immersing their body or head, respectively.

We used logistic regression to model the effect of swimming and water quality on illness. Models included continuous measures of water quality as predictor variables and a 0/1 indicator of illness as the outcome. We used nested interaction terms to allow contrasts among swimmers and between swimmers and nonswimmers. To evaluate the overall risk associated with swimming, we excluded the water-quality measures from the models. We determined odds ratios (ORs) by taking the exponent of the regression coefficients from the logistic regression models. We estimated adjusted predicted probabilities from logistic regression models, holding covariates constant at their mean.

Variables that were related to GI illness or swimming in tabulations, or were suspected by investigators to correlate with GI illness, were considered for regression models. As a result, we evaluated the following variables in initial models: age; sex; race; allergies; swimming within 48 hr before the beach visit or between the beach visit and telephone interview; contact with animals; contact with persons with GI illness; consumption of raw meat, fish, or undercooked eggs; presence of chronic GI illness, skin conditions, or asthma; frequency of beach visits; and use of nose plugs. We excluded from the analysis beachgoers who reported any GI symptoms within 48 hr of the beach visit.

We selected final regression models using backward deletion as described by Rothman and Greenland (1998). Initially, all covariates were included in the model. Covariates were then removed in an iterative fashion until removal of any remaining covariates resulted in > 5% change in the exposure–illness relationship.

We used SAS, version 8.0 (SAS Institute, Cary, NC), S-plus, version 6.1 (Insightful Corp. 2002), and Stata, version 8.2 (Stata Corp., College Station, TX) for data analysis.

## Results

We interviewed beachgoers at beach A from 1 June 2003 through 3 August 2003 on weekends and holidays, for a total of 20 days. We interviewed beachgoers at beach B from 2 August 2003 through 14 September 2003, for a total of 13 days. At beach B, no interviews were conducted because of bad weather on 17 August and 1 September. There were 5,796 household interview attempts at both beaches. The household interviewing response rate (completed/attempted) through the completion of the telephone interview was 56%. Data were available for a total of 3,221 households (5,717 individuals), 1,639 households (2,840 individuals) at the Lake Erie beach (beach B), and 1,582 households (2,877 individuals) at the Lake Michigan beach (beach A). After excluding subjects with GI illness at baseline, data were available for 5,667 individuals.

### Water quality.

QPCRCE results for the measurements of indicator organisms on study days are shown in Table 1. The QPCRCE for *Bacteroides* was considerably higher than that for *Enterococcus*, although there were more results below the LOD for *Bacteroides*. At beach A, 28% of *Bacteroides* samples were below the LOD, and at beach B, 21% of *Bacteroides* samples were below the LOD. *Enterococcus* QPCRCE at beach A was slightly higher than at beach B (*p* = 0.06). There was no difference in *Bacteroides* QPCRCE between beach A and beach B.

### Swimming and GI illness.

The incidence of GI illness among swimmers and nonswim-mers is shown in Table 2. At beach A, the incidence of GI illness was 10% among swimmers, compared to 5% among nonswimmers. At beach B, the incidence among swimmers ranged from 12% for those with any contact with water and to 14% for those who immersed their head, compared to 10% in nonswimmers. Fewer beachgoers reported swimming at beach B than at beach A: at beach A, 75% of respondents reported contact with water, whereas only about 50% reported contact with water at beach B. GI illness was associated with swimming at both beaches. At beach A, those with any contact with water were almost twice as likely to have GI illness compared with nonswimmers [adjusted OR (AOR) = 1.96; 95% confidence interval (CI), 1.33–2.90]. Those immersing their body and head were at slightly higher risk (for body immersion: AOR = 2.26; 95% CI, 1.51–3.39; for head immersion: AOR = 2.14; 95% CI, 1.41–3.27). The risk of GI illness associated with swimming was slightly less at beach B (for head immersion: AOR = 1.50; 95% CI, 1.06–2.13).

At both beaches, swimmers were younger, more likely to be male, more likely to eat food or consume beverages at the beach, and more likely to report allergies. At beach A, swimmers were more likely to have consumed raw or undercooked meat within 48 hr of the beach visit, more likely to have had contact with known or unknown animals, and slightly less likely to report chronic GI illness (1.2% vs. 2.2%). At beach B, nonswimmers were more likely to have GI symptoms at baseline (3.4% vs. 1.7%) and more likely to report asthma.

### Water quality and GI illness.

Table 3 shows the associations between QPCRCE and the risk of GI illness for each beach and both beaches combined. In these models, contrasts were created to show ORs of a unit increase in exposure among swimmers. At both beaches, we observed a trend between increasing mean log_10_ QPCRCE of *Enterococcus* and risk of GI illness.

We observed a slightly stronger association with GI illness for the overall daily average of *Enterococcus* QPCRCE than for averages specific to a beachgoer’s reported swimming location. At beach A, a log_10_ increase in the daily average of *Enterococcus* QPCRCE was associated with a 1.43 (95% CI, 1.08–1.90) increase in the odds of GI illness for those immersing their bodies. At beach B, estimates for trends between GI illness and *Enterococcus* QPCRCE daily averages were also elevated but slightly lower.

*Bacteroides* QPCRCE was positively associated with illness at beach B, but trends were of borderline statistical significance (*p* < 0.1). Again, we found little difference between the overall daily average and averages based on a beachgoer’s reported swimming location. No association was observed between *Bacteroides* QPCRCE and GI illness at beach A.

Trends tended to be stronger when we defined swimming as body or head immersion than when we defined swimming as any contact with water. Defining swimming as head immersion at beach B resulted in a weaker trend than did body immersion or any contact with water, but at this beach only 18% of respondents reported immersing their head.

We included an indicator for beach in the models that combined the results for both beaches. No trend between GI illness and *Bacteroides* QPCRCE was observed when both beaches are combined because of the lack of an observed trend at beach A. Trends between illness and daily averages of *Enterococcus* QPCRCE were statistically significant (*p* = 0.005). A log_10_ increase in *Enterococcus* QPCRCE was associated with a 1.37 (95% CI, 1.10–1.71) increase in the odds of GI illness. A likelihood ratio test comparing the saturated model with the restricted model indicated that the interaction between beach and daily averaged water-quality measure was not statistically significant (*p* = 0.48). The beach effect was statistically significant (AOR = 0.64; 95% CI, 0.52–0.73, beach B vs. beach A), reflecting the lower overall incidence of GI illness at beach A.

**Figure 1:**
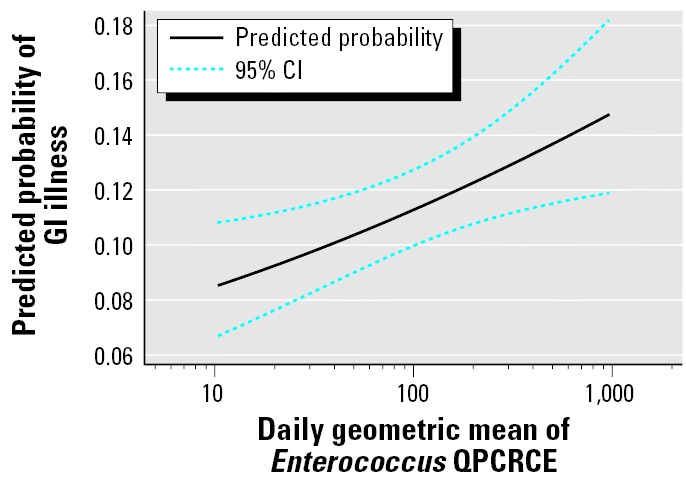
Predicted probabilities of GI illness as a function of *Enterococcus* QPCRCE, predicted from the logistic regression model, adjusted for age and beach.

Figure 1 illustrates the predicted probabilities for GI illness as a function of the log_10_ QPCRCE *Enterococcus* measures for swimmers immersing their bodies at both beaches combined.

We examined the 0800 hr samples separately to see if water samples tested in the morning were predictive of GI illness among swimmers that day. As shown in Table 3, *Enterococcus* QPCRCE measured at 0800 hr was associated with GI illness that day. Although the trends are not as strong as the daily or location-specific averages, *Enterococcus* QPCRCE measured at 0800 hr was predictive of GI illness that day, with a log_10_ increase associated with an approximately 1.2 increase in the odds of GI illness.

The trend between increasing *Enterococcus* QPCRCE with illness was stronger among swimmers who spent more time in the water (Table 4). A log_10_ increase in *Enterococcus* QPCRCE and GI illness among those spending > 2 hr in the water was associated with a nearly 3-fold increase in the odds of GI illness (AOR = 2.89; 95% CI, 1.55–5.40).

## Discussion

This is the first study to demonstrate the ability of rapid indicator methods to predict health effects. The results showed that *Enterococcus* measured by QPCR can predict GI illness after swimming in fecally contaminated fresh water. The results also demonstrate that samples collected each morning could allow beach managers to assess the microbiological safety of the beach before most beachgoers are exposed. Incorporation of rapid measurements such as these into a regulatory framework has the potential to improve beach management decisions and protect swimmers’ health.

Swimmers at the two Great Lake beaches had a higher incidence of GI illness than did nonswimmers. Among swimmers at beach A, risk of illness increased as daily averages of *Enterococcus* QPCRCE increased. Among swimmers at beach B, daily averages of *Enterococcus* QPCRCE were also positively associated with GI illness, although the 95% CI of the OR included 1.0. This power to detect a significant effect at beach B may have been limited because of fewer swimmers at this beach. Combining beaches produced significant trends with both daily averages and averages of samples collected at 0800 hr only. The association between *Enterococcus* QPCRCE and GI illness strengthened as the time spent in water increased, possibly reflecting an increased risk of illness resulting from increased exposure to fecal contamination among those spending longer periods in the water.

Using QPCRCE averages specific to a beachgoer’s reported swimming location did not improve the relationship between illness and water quality. This may be because swimmers swam in several locations and did not restrict their swimming along one transect. Also, recall or reporting errors in swimming location would lead to misclassification. As a result, the daily averages that combined results at each location, time, and water depth may have been a better characterization of the exposure of an average swimmer.

Results for *Bacteroides* QPCRCE were less promising, and interpretation of the results is limited because a relatively high proportion of samples were below the LOD. Although a borderline trend was noted at beach B, where fewer samples were below the LOD, no trend was observed at beach A. Imputing the censored values using one-half the LOD did not improve the relationship, nor did eliminating the censored data points. Efforts are being made to improve the sensitivity of the *Bacteroides* assay with the hope of improving its reliability as a predictor of illness. One of the advantages of the QPCR method is the ability to archive samples, and if improvements are made to the assay, they will be retested.

The two beaches differed with respect to swimming, demographic characteristics, and baseline illness. At beach B, more respondents were > 35 years of age (59% vs. 39%) and white (90% vs. 73%) than at beach A. A higher proportion of nonswimmers at beach B reported illness than at beach A (10% vs. 5%). Differences in the study populations may have been responsible for the higher overall risk in illness among swimmers compared with non-swimmers at beach A.

We observed no striking difference in the trend between illness and water quality for the different types of swimming definitions. With the exception of *Enterococcus* at the Lake Erie beach (beach B), trends tended to be stronger when swimming was defined as body immersion and head immersion compared with any contact with water. This is consistent with the hypothesis that more active types of swimming would result in greater exposure to fecally contaminated water.

Because trends were evaluated among swimmers, it is unlikely that the observed associations could be attributed to unmeasured confounding factors. It is unlikely that swimmers associated themselves with different water quality with respect to characteristics that could affect GI illness. Adjusting for covariates tended to strengthen the trend and association between illness and water quality.

Although we selected beaches affected by human fecal contamination, we do not know whether fecal contamination from other bathers was an important contributor to the overall level of fecal contamination. Although there was no significant difference in *Enterococcus* QPCRCE by collection time, the average QPCRCE increased slightly throughout the day, suggesting that swimmers may have contributed some fecal contamination.

Because QPCR relies on DNA to quantify organisms, viable organisms are not necessary for measurement. As a result, indicators measured by QPCR may differ in their sensitivity to some environmental conditions. For example, we did not see a reduction in QPCRCE over the course of the day, an effect that has been observed for culture-based indicator organisms resulting from die-off caused by ultraviolet radiation (Whitman et al. 2004). There is a need for additional studies to better understand how indicators measured by QPCR are affected by physical and environmental factors in recreational waters.

Because this is the first and only study to evaluate the ability of rapid water-quality indicators to predict GI illness, additional studies will be required to evaluate the generalizability of these findings. Additional studies and analyses will help determine whether these preliminary findings are consistent and robust enough from a regulatory perspective to recommend a rapid indicator for recreational water quality, and to evaluate the conditions under which such indicators can successfully be applied. Ultimately, the use of faster indicators of recreational water quality will result in the ability to make decisions about recreational water quality on the day of sample collection. This, in turn, could lower GI illnesses in communities, especially in those dependent on beach-related tourism.

## Figures and Tables

**Table 1:** Summary statistics for log_10_ indicator organisms, measured by QPCR.

	*Enterococcus*^a^		*Bacteroides*^b^	
	Beach A	Beach B	Beach A	Beach B
No. of days	20	13	20	13
No. of samples	329	350	329	350
QPCRCE/100 mL				
Mean	2.04	1.90	3.08	3.02
Median	2.07	2.05	3.34	3.63
SD	0.97	1.03	1.12	1.56
Minimum/maximum	–1.53/4.20	–1.75/4.17	0.97/5.37	–0.23/5.57
No. (%) < LOD	9 (2.74)	11 (3.14)	91 (27.66)	74 (21.14)

a *p* = 0.06 for difference in log QPCRCE (*t*-test).

b *p* = 0.55 for difference in log QPCRCE (*t*-test).

**Table 2:** GI illness among swimmers and nonswimmers.

	No. (% of total)	No. reporting GI illness (% of exposed)	AOR (95% CI)
Beach A			
No contact with water	722 (25)	36 (5.0)	
Any contact with water	2,154 (75)	208 (9.7)	1.96 (1.33–2.90)*
Body immersion	1,667 (58)	169 (10)	2.26 (1.51–3.39)*
Head immersion	1,210 (42)	117 (9.7)	2.14 (1.41–3.27)*
Total respondents	2,876^a^		
Beach B			
No contact with water	1,535 (54)	147 (10)	
Any contact with water	1,305 (46)	159 (12)	1.27 (0.97–1.67)**
Body immersion	757 (27)	101 (13)	1.45 (1.06–1.98)*
Head immersion	524 (18)	71 (14)	1.50 (1.06–2.13)*
Total respondents	2,840		
Both beaches			
No contact with water		183 (8)	
Any contact with water		367 (11)	1.45 (1.17–1.80)*
Body immersion		270 (11)	1.63 (1.29–2.07)*
Head immersion		188 (11)	1.61 (1.25–2.07)*

a One missing value.

* *p* < 0.05.

** *p* < 0.1.

**Table 3.** AORs (95% CIs) for a 1 log_10_ increase in *Enterococcus* QPCRCE and GI illness.^a^

	*Enterococcus* QPCRCE			*Bacteroides* QPCRCE		
Exposure	Average by day	Average by day and location of swimming	Average 0800-hr sample	Average by day	Average by day and location of swimming	Average 0800-hr sample
Beach A						
Any contact	1.36* (1.05–1.76)	1.32* (1.04–1.67)	—	0.89 (0.75–1.06)	0.87 (0.74–1.02)	—
Body immersion	1.43* (1.08–1.90)	1.34* (1.03–1.74)	—	0.89 (0.74–1.07)	0.87 (0.73–1.04)	—
Head immersion	1.49* (1.07–2.08)	1.41* (1.04–1.90)	—	0.84 (0.67–1.05)	0.87 (0.37–1.17)	—
Beach B						
Any contact	1.25 (0.93–1.67)	1.20 (0.87–1.66)	—	1.15 (0.94–1.41)	1.12** (0.97–1.31)	—
Body immersion	1.38** (0.94–2.01)	1.27 (0.90–1.81)	—	1.24** (0.96–1.60)	1.17** (0.97–1.40)	—
Head immersion	1.17 (0.76–1.82)	1.15 (0.77–1.71)	—	1.28** (0.95–1.73)	1.20* (0.97–1.49)	—
Beaches A and B combined						
Any contact	1.30* (1.08–1.57)	1.25* (1.05–1.49)	1.18* (1.03–1.34)	0.99 (0.87–1.13)	1.01 (0.90–1.12)	0.95 (0.86–1.05)
Body immersion	1.37* (1.10–1.71)	1.26 (1.04–1.53)	1.21* (1.04–1.40)	1.00 (0.86–1.16)	1.01 (0.89–1.14)	0.94 (0.84–1.06)
Head immersion	1.35* (1.05–1.75)	1.29* (1.03–1.63)	1.21* (1.02–1.44)	0.99 (0.83–1.17)	1.00 (0.86–1.15)	0.92 (0.80–1.06)

a ORs estimated from multivariate logistic regression of GI illness on the log (base 10) indicator measure.

* *p* < 0.05.

** *p* < 0.1.

**Table 4:** AORs (95% CIs) for a 1 log_10_ increase in the daily average of *Enterococcus* QPCRCE and GI illness among swimmers by time spent in water, beaches A and B combined.^a^

Time spent in water (min)	No.	AOR per 1 log_10_ increase (95% CI)
≥ 15	2,477	1.45 (1.14–1.85)*
≥ 30	1,572	1.48 (1.12–1.96)*
≥ 60	735	1.84 (1.25–2.72)*
≥ 120	289	2.89 (1.55–5.40)*

a Body immersed in water.

* *p* < 0.05.
